# A Large Retrospective Assessment of Voriconazole Exposure in Patients Treated with Extracorporeal Membrane Oxygenation

**DOI:** 10.3390/microorganisms9071543

**Published:** 2021-07-20

**Authors:** Ruth Van Daele, Britt Bekkers, Mattias Lindfors, Lars Mikael Broman, Alexander Schauwvlieghe, Bart Rijnders, Nicole G. M. Hunfeld, Nicole P. Juffermans, Fabio Silvio Taccone, Carlos Antônio Coimbra Sousa, Luc-Marie Jacquet, Pierre-François Laterre, Eric Nulens, Veerle Grootaert, Haifa Lyster, Anna Reed, Brijesh Patel, Philippe Meersseman, Yves Debaveye, Joost Wauters, Christophe Vandenbriele, Isabel Spriet

**Affiliations:** 1 Department of Pharmaceutical and Pharmacological Sciences, KU Leuven, 3000 Leuven, Belgium; isabel.spriet@uzleuven.be; 2 Pharmacy Department, University Hospitals Leuven, 3000 Leuven, Belgium; britt.bekkers@uzleuven.be; 3 ECMO Centre Karolinska, Department of Pediatric Perioperative Medicine and Intensive Care, Karolinska University Hospital, 17177 Stockholm, Sweden; mattias.lindfors@sll.se (M.L.); lars.broman@sll.se (L.M.B.); 4 Department of Physiology and Pharmacology, Karolinska Institutet, 17177 Stockholm, Sweden; 5 Department of Hematology, Ghent University Hospital, 9000 Ghent, Belgium; Alexander.Schauwvlieghe@uzgent.be; 6 Department of Internal Medicine, Section of Infectious Diseases, Erasmus University Medical Center, 3015 CP Rotterdam, The Netherlands; b.rijnders@erasmusmc.nl; 7 Department of Medical Microbiology and Infectious Diseases, Erasmus University Medical Center, 3015 CP Rotterdam, The Netherlands; 8 Department of Intensive Care and Department of Hospital Pharmacy, Erasmus University Medical Center, 3015 CP Rotterdam, The Netherlands; n.hunfeld@erasmusmc.nl; 9 Department of Intensive Care, Amsterdam University Medical Center, 1105 AZ Amsterdam, The Netherlands; n.p.juffermans@amc.uva.nl; 10 Department of Intensive Care, Hôpital Erasme, Université Libre de Bruxelles (ULB), 1050 Brussels, Belgium; ftaccone@ulb.ac.be (F.S.T.); Carlos.sousa85@gmail.com (C.A.C.S.); 11 Cardiovascular Intensive Care, Cliniques Universitaires Saint-Luc, 1050 Brussels, Belgium; luc-marie.jacquet@uclouvain.be; 12 Department of Intensive Care, Cliniques Universitaires St-Luc, Université Catholique de Louvain, 1050 Brussels, Belgium; pierre-francois.laterre@uclouvain.be; 13 Laboratory Medicine, Medical Microbiology, Algemeen Ziekenhuis Sint-Jan, Brugge-Oostende, 8000 Brugge, Belgium; eric.nulens@azsintjan.be; 14 Pharmacy Department, Algemeen Ziekenhuis Sint-Jan Brugge-Oostende AV, 8000 Brugge, Belgium; veerle.grootaert@azsintjan.be; 15 Pharmacy Department, Royal Brompton & Harefield Hospitals, London SW3 6NP, UK; H.Lyster@rbht.nhs.uk; 16 Cardiothoracic Transplant Unit, Royal Brompton & Harefield Hospitals, London SW3 6NP, UK; A.Reed@rbht.nhs.uk; 17 Imperial College London, London SW3 6NP, UK; 18 Division of Anaesthetics, Pain Medicine & Intensive Care, Department of Surgery & Cancer, Faculty of Medicine, Imperial College, London SW3 6NP, UK; brijesh.patel@imperial.ac.uk; 19 Department of Adult Intensive Care, The Royal Brompton and Harefield Hospitals, London SW3 6NP, UK; christophe.vandenbriele@uzleuven.be; 20 Department of General Internal Medicine, Medical Intensive Care Unit, University Hospitals Leuven, 3000 Leuven, Belgium; philippe.meersseman@uzleuven.be; 21 Department of Cellular and Molecular Medicine, KU Leuven, 3000 Leuven, Belgium; yves.debaveye@uzleuven.be; 22 Intensive Care Unit, University Hospitals Leuven, 3000 Leuven, Belgium; 23 Department of Microbiology and Immunology, KU Leuven, 3000 Leuven, Belgium; joost.wauters@uzleuven.be; 24 Medical Intensive Care Unit, University Hospitals Leuven, 3000 Leuven, Belgium; 25 Department of Cardiovascular Sciences, KU Leuven, 3000 Leuven, Belgium; 26 Department of Cardiovascular Diseases, University Hospitals Leuven, 3000 Leuven, Belgium

**Keywords:** invasive fungal infections, critically ill patients, voriconazole, exposure, pharmacokinetics, variability, extracorporeal membrane oxygenation, therapeutic drug monitoring

## Abstract

Background: Voriconazole is one of the first-line therapies for invasive pulmonary aspergillosis. Drug concentrations might be significantly influenced by the use of extracorporeal membrane oxygenation (ECMO). We aimed to assess the effect of ECMO on voriconazole exposure in a large patient population. Methods: Critically ill patients from eight centers in four countries treated with voriconazole during ECMO support were included in this retrospective study. Voriconazole concentrations were collected in a period on ECMO and before/after ECMO treatment. Multivariate analyses were performed to evaluate the effect of ECMO on voriconazole exposure and to assess the impact of possible saturation of the circuit’s binding sites over time. Results: Sixty-nine patients and 337 samples (190 during and 147 before/after ECMO) were analyzed. Subtherapeutic concentrations (<2 mg/L) were observed in 56% of the samples during ECMO and 39% without ECMO (*p* = 0.80). The median trough concentration, for a similar daily dose, was 2.4 (1.2–4.7) mg/L under ECMO and 2.5 (1.4–3.9) mg/L without ECMO (*p* = 0.58). Extensive inter-and intrasubject variability were observed. Neither ECMO nor squared day of ECMO (saturation) were retained as significant covariates on voriconazole exposure. Conclusions: No significant ECMO-effect was observed on voriconazole exposure. A large proportion of patients had voriconazole subtherapeutic concentrations.

## 1. Introduction

Invasive mold infections are life-threatening diseases, associated with a high morbidity and mortality, and are frequently caused by *Aspergillus spp*. [1,2,3,4,5]. The AspICU-study showed that critically ill patients are at-risk to develop these infections [6]. More recently, severe viral infections, such as influenza and COVID-19, have been associated with invasive pulmonary aspergillosis (IPA) in critically ill patients, even in those without pre-existing comorbidities [5,7,8,9].

Critical illness is often associated with pathophysiological changes, potentially impacting the pharmacokinetics (PK) of administered drugs. Capillary leak and fluid shifts might occur and the plasma protein binding of drugs can be affected due to hypoalbuminemia, all that may lead to an increased volume of distribution (Vd). Besides, the renal and liver function might be impaired as well, hampering drug metabolism and clearance. In contrast, some patients present with augmented renal clearance (ARC), which might result in excessive drug elimination and insufficient drug exposure [10]. In a subset of intensive care unit (ICU) patients, extracorporeal membrane oxygenation (ECMO) is used to allow cardio-pulmonary support in a situation of life-threatening respiratory and/or cardiac failure [11,12]. Due to the increasing use of ECMO in adult ICU patients since the 2009 H1N1 influenza and currently the COVID-19 pandemics, its influence on drug exposure has become a matter of interest, as ECMO might as well alter the PK of different drugs [8,10,12,13,14]. Priming of the ECMO circuit, typically carried out with crystalloid solutions, can increase the Vd, particularly for water-soluble molecules. In addition, predominantly lipophilic drugs potentially bind to the artificial components of the extracorporeal circuit [7,11,12,13]. However, adsorption to the binding sites seems to be saturable, leading to significant drug loss in a new circuit and a reduced drug loss over time [12]. The physicochemical properties of a drug, e.g., the degree of lipophilicity expressed as the octanol/water partition coefficient (logP), determine the effect of ECMO on drug exposure [10,12,15].

Voriconazole, a broad-spectrum triazole antifungal drug, is currently recommended as one of the first-line therapies for IPA [1,3,4]. A clear exposure-response relationship has been established, with recommended target trough concentrations higher than 1–2 mg/L and lower than 5–6 mg/L [1]. Exposure to voriconazole is influenced by many factors, such as non-linear PK, involvement in drug-drug interactions (DDIs), erratic absorption and continuous renal replacement therapy (CRRT), leading to a high intra- and interpatient variability, which is even more pronounced in critically ill patients [1,16,17].

It has been assumed that voriconazole exposure is impacted by ECMO therapy. However, this insight is only based on three ex vivo studies and six case reports in which it was suggested that voriconazole sequestrates to the extracorporeal circuit, given its relative lipophilicity (log *p* value of 2.56) [11,12,15,18,19,20,21,22,23]. The ex vivo studies reported voriconazole losses even up to 80% at the end of a 24 h study period [11,21,22]. These results were supported by multiple case reports [12,15,18,19,20,23]. Undetectable voriconazole concentrations and difficulties to attain therapeutic exposure under ECMO were reported by Ruiz et al. and Mathieu et al. [15,18]. Saturation of the circuit’s binding sites was suggested by Spriet et al. who increased the voriconazole dose at the moment of ECMO initiation to successfully avoid subtherapeutic concentrations but after a few days, drug accumulation was observed. Saturation was also indicated by Winiszewski et al. who observed drops in voriconazole concentration at introduction of ECMO and after each ECMO membrane change [12,23]. This in contrast to the case report by Peterson et al., in which a continued need for high voriconazole doses under ECMO was reported with only decreasing voriconazole requirements after ECMO decannulation [20].

Based on previously published reports, it seems that therapeutic plasma concentrations of voriconazole during ECMO cannot be guaranteed but that the impact of ECMO on the plasma levels is not fully understood. We aimed to assess the effect of ECMO on voriconazole systemic exposure in a large multicenter, retrospective study in order to evaluate the need for dose adjustments in function of the duration of ECMO.

## 2. Materials and Methods

### 2.1. Study Design, Population and Setting

A retrospective, multicenter study was conducted in eight hospitals, i.e., four Belgian hospitals (University Hospitals Leuven, which was the primary research site; AZ Sint-Jan Brugge-Oostende; Université Catholique de Louvain; Université Libre de Bruxelles), two Dutch hospitals (Amsterdam University Medical Center and Erasmus University Medical Center Rotterdam), one Swedish hospital (Karolinska University Hospital) and one hospital from the United Kingdom (Royal Brompton and Harefield Hospitals) between 1 January 2009 and 31 July 2020. Ethical approval was obtained by the local ethics committees in each study site or was waived in case the use of retrospective patient data generated during routine care did not fall under the local research legislation.

All adult patients hospitalized in the ICU, who were treated with voriconazole and simultaneously received ECMO-support during at least a part of this antifungal treatment, were eligible for inclusion in the study. Patients could only be included when at least one voriconazole trough concentration was available during ECMO-support. There were no restrictions in function of medical indication, neither in function of installed voriconazole dose. As control group, voriconazole trough concentrations obtained in the same patients before or after ECMO treatment were used.

### 2.2. Data Sources and Collection

The following information was collected: patient’s demographics (gender, age, body weight, length of hospital and ICU stay), renal replacement therapy (intermittent hemodialysis (IHD) or continuous renal replacement therapy (CRRT)), severity of critical illness (APACHE II and SOFA scores), biochemical parameters (C-reactive protein (CRP), bilirubin (total and direct), gamma glutamyltransferase (GGT), alkaline phosphatase (APT), aspartate transaminase (AST), alanine transaminase (ALT), lactate dehydrogenase (LDH)), voriconazole administration (timing and dosing information, voriconazole trough concentrations) and concomitant administration of possible interacting drugs, such as proton pump inhibitors (PPIs) and cytochrome *p* 450 (CYP450)-inducers (rifampicin, rifabutin, phenytoin, phenobarbital, carbamazepine, St. John’s wort, antiretrovirals).

Data collection concerning ECMO comprised details on the type of oxygenator and blood pump and the duration of ECMO versus timing of voriconazole blood sampling. Each time the ECMO circuit or one of its components (i.e., oxygenator, cannula, tubing) was changed, the ECMO-day was reset to day 1, since new binding sites were presumed to be available.

### 2.3. Statistical Analysis

The primary outcome variable was the voriconazole trough concentration, both as continuous and categorical variable. For the latter, the categories were defined as subtherapeutic (<2 mg/L) versus adequate (>2 mg/L) concentrations. The reported concentrations were considered as actual trough concentrations if they were collected 12 ± 1 h after the previous administered dose (sample set A). However, subtherapeutic concentrations that were collected too early (<11 h after previous dose) or supratherapeutic concentrations (>5.5 mg/L) that were collected too late (>13 h after previous dose), were included additionally in the analyses with voriconazole as categorical variable, since classification of these concentrations would not have changed in case of correct sampling time (sample set B). The effect of ECMO was determined using generalized estimating equation (GEE) analysis. The presence of ECMO was defined as the association of ECMO within 24 h before sampling (binomial variable). In order to evaluate ECMO as an independent covariate impacting voriconazole trough exposure, other previously identified relevant covariates were also taken into account in the multivariate analysis, i.e., severity of illness (APACHE II score), information concerning voriconazole administration (voriconazole daily dose (mg/kg during previous 24 h), day of voriconazole therapy, mode of administration), liver function (GGT), inflammation (CRP), presence of CRRT and DDIs (PPI or inducer). Moreover, to ensure that all covariates that might influence voriconazole concentrations were included, additional covariates were tested in univariate analyses and, if found to be significantly associated, included in the multivariate analysis: hospital, age, gender, body weight (BW), presence of loading dose, IHD and biochemical parameters on the day of sampling (total bilirubin, direct bilirubin, APT, AST, ALT, LDH). After inclusion of all relevant covariates, a backward selection was performed until a final model with only significantly associated parameters was attained or until ECMO was excluded from the model as non-significantly associated parameter.

To study the presence of circuit saturation, ECMO as binomial predictor was replaced by the squared day of ECMO. The squared day of ECMO was used instead of the day of ECMO as such, since the relationship between the day of ECMO and the voriconazole trough exposure was not expected to be linear but rather follow a squared function with a decrease in voriconazole exposure just after ECMO initiation and an increase after a few days of ECMO due to saturation of the circuit. The same methodology as above (multivariate analysis using GEE modeling with backward selection) was used but only for the subset of voriconazole concentrations that were sampled while on ECMO.

For statistical analysis, missing continuous data were completed with the median value for the same patient, if available, or the median of the total population. To compare the descriptive characteristics between the samples with versus without ECMO, a univariate GEE analysis was used with ECMO as binomial outcome variable. The significance level was set to 0.05. Statistical analyses were carried out with R statistics (R version 3.6.3; The R Foundation for Statistical Computing, Vienna, Austria).

## 3. Results

### 3.1. Patient Characteristics

During the study period, 69 patients were included (Table S1). The median (IQR) age was 54 (42–60) years and 67% were male. Patients’ median weight was 77 (65–95) kg. The length of ICU stay ranged from 8 to 228 days (median 40 days) and 33/69 (48%) of the patients died during their ICU stay. On admission, the median APACHE II score was 18 (14–23).

### 3.2. Extracorporeal Membrane Oxygenation

As shown in Table 1, veno-venous (VV) ECMO was used in the majority (74%) of patients; 10% was on veno-arterial (VA) ECMO and the remaining patients (16%) switched between VV and VA-ECMO during ECMO support. The median duration of ECMO therapy was 19 (11–33) days. A change in circuit or one of its components was performed during ECMO treatment in 22 (31.9%) patients.

### 3.3. Voriconazole Concentrations

During voriconazole therapy a total of 474 trough samples were collected. When excluding all samples that deviated more than 1 h from the theoretical sampling time (<11 h or >13 h postdose), 282 samples were included, of which 145 sampled during ECMO and 137 before or after ECMO (sample set A) (Table 2). When classifying voriconazole concentrations in subtherapeutic versus adequate exposure subgroups, the subtherapeutic concentrations that were collected too early (<11 h after previous dose) or supratherapeutic concentrations (>5.5 mg/L) that were collected too late (>13 h after previous dose), were added, which resulted in 337 samples of which 190 with ECMO and 147 before or after ECMO (sample set B) (Table 2). Table S2 reports the number of voriconazole samples collected per participating center.

**Figure 1:**
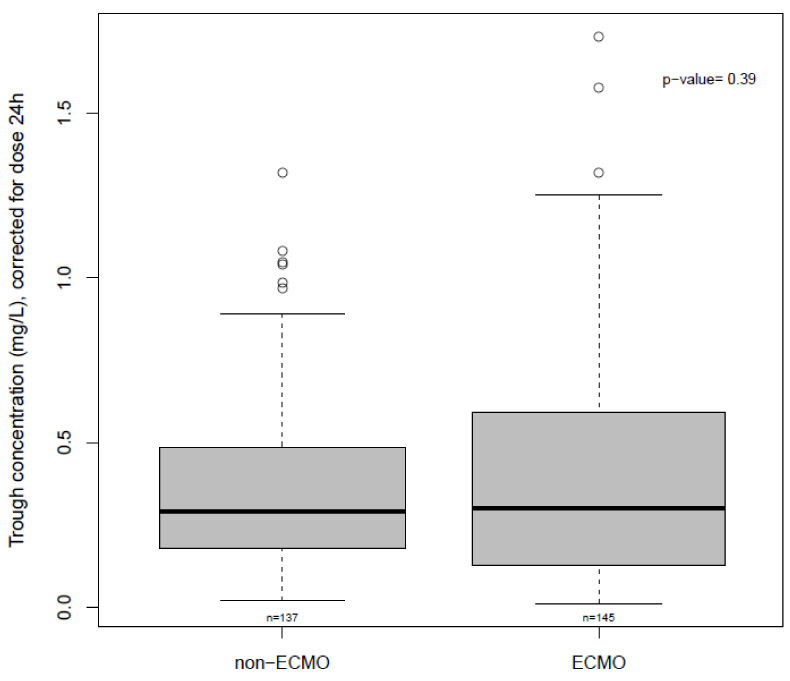
Boxplot; Voriconazole trough concentrations, corrected for dose 24 h, on ECMO versus non-ECMO sampling days.

**Figure 2:**
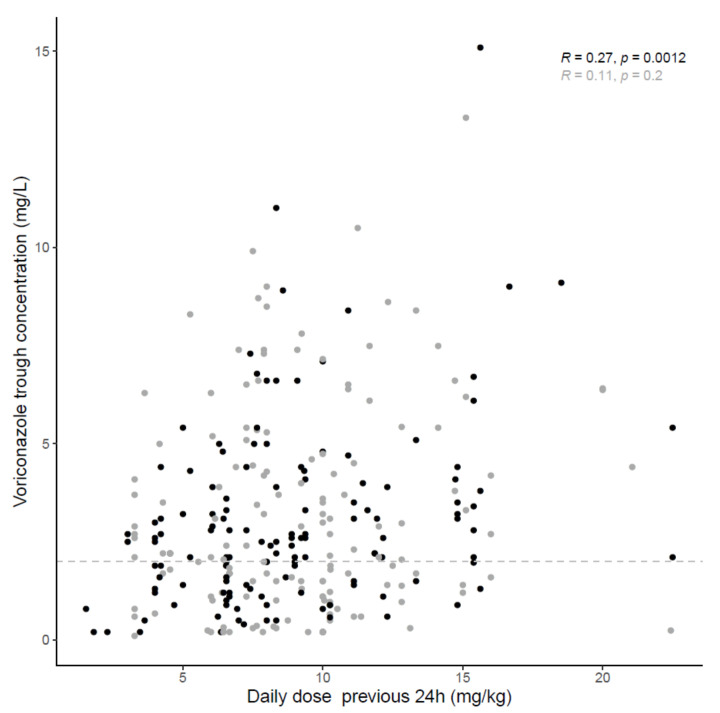
Scatterplot; Voriconazole concentrations on ECMO (grey) versus non-ECMO (black) sampling days with spearman-coefficient and *p*-value (*n* = 282). The dashed line represents the lower limit concentration of 2 mg/L.

**Figure 3:**
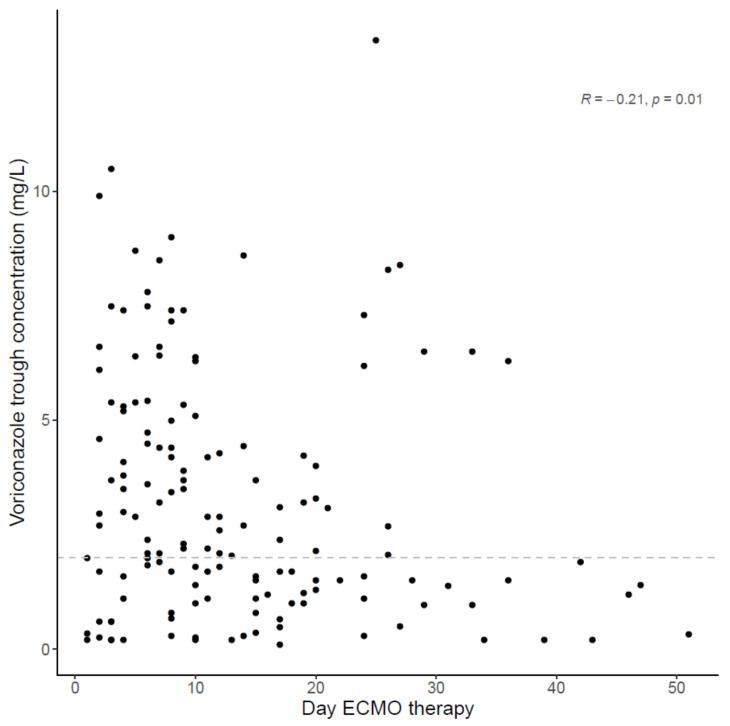
Voriconazole trough concentration in function of the day of ECMO with spearman-coefficient and *p*-value (*n* = 145). The dashed line represents the lower limit concentration of 2 mg/L.

The median daily voriconazole dose on ECMO versus non-ECMO sampling days was 9.2 (6.7–10.9) mg/kg and 8.1 (6.5–11.1) mg/kg, respectively (*p* = 0.76). The median trough concentration was 2.4 (1.2–4.7) mg/L under ECMO and 2.5 (1.4–3.9) mg/L on non-ECMO sampling days (*p* = 0.58). Voriconazole trough concentrations (corrected for the administered dose 24 h previously to sampling) on ECMO and non-ECMO sampling days are depicted in Figure 1. In Figure 2 and Figure 3, trough concentrations are shown in function of the administered daily dose and the day of ECMO, respectively. In the Supplementary Data, Figure S1 shows the voriconazole trough concentration for three different timeframes (pre, during and post ECMO) and Figure S2, the voriconazole dose in function of the day of ECMO is depicted.

Subtherapeutic voriconazole concentrations were observed in 56% of samples during ECMO and 39% of non-ECMO samples (*p* = 0.80). Inter- and intrasubject variability (%CV) in the voriconazole trough concentration (corrected for the dose) were 47% and 78% on sampling days under ECMO and 46% and 60% for non-ECMO sampling.

### 3.4. Generalized Estimating Equation (GEE) Analyses

None of the additionally tested covariates in the univariate analysis were significantly associated with the trough concentrations (continuous variable). The multivariate analyses in which voriconazole exposure was expressed as continuous concentration, were performed with both the presence of ECMO as binomial predictor (taking into account all voriconazole trough concentrations (*n* = 277)) and with the squared day of ECMO (saturation effect) in the subgroup of voriconazole concentrations measured under ECMO (*n* = 140). In both analyses both the presence of ECMO and the ECMO day were excluded through backward selection. Hence, neither the presence of ECMO nor the squared ECMO day were independently associated with voriconazole exposure.

When using the trough concentration as categorical variable, patient’s age and participating hospital were retained in the univariate analysis and included for further analysis. Multivariate analyses with voriconazole exposure as a categorical outcome parameter were also performed with both the presence of ECMO as binomial predictor (taking into account all voriconazole trough concentrations (*n* = 322)) and with the squared day of ECMO (saturation effect) in the subgroup of voriconazole concentrations measured under ECMO (*n* = 175). Again, in both analyses, neither the presence of ECMO nor the squared ECMO day were found to be significantly associated with voriconazole exposure as both were excluded through backward selection.

## 4. Discussion

This large, retrospective study could not demonstrate a significant impact of ECMO on voriconazole exposure. However, a large proportion of subtherapeutic concentrations were observed, both in the ECMO and non-ECMO treatment period.

Similar median doses of voriconazole on ECMO and non-ECMO days resulted in similar voriconazole concentrations, refuting the need for higher voriconazole administration doses during ECMO-therapy. Voriconazole trough exposure was highly variable and overlapping on ECMO versus non-ECMO sampling days. These results were confirmed in multivariate analysis, in which neither the presence of ECMO nor the squared day of ECMO treatment were identified as parameters significantly associated with voriconazole exposure. No clear saturation effect over time could be observed.

Literature describing the exposure to voriconazole during ECMO is limited to three ex vivo studies and six case reports [11,12,15,18,19,20,21,22,23]. The ex vivo studies demonstrated significant voriconazole losses in ECMO circuits, even up to 80% in some reports [11,21,22]. However, in these studies, patient and disease related factors, such as metabolism, elimination, inflammatory mediators, altered pH and concomitant drugs, were not accounted for [21,22]. Moreover, these studies looked at a limited period of time (24 h) after addition of only one single voriconazole dose [11,18,21,22]. Low voriconazole exposure and a need for dose augmentation during ECMO were also described in the case reports. However, alternative causes, often mentioned in the limitation sections of the reports, might also explain this low exposure. The increase in voriconazole trough concentrations in the case report by Spriet et al. might also be explained by the time needed to reach steady state after dose augmentation instead of saturation of the ECMO circuit [12]. In the case report of Winiszewski et al. sudden drops in voriconazole concentrations were also observed in the period between membrane changes [23]. In the case report of Ruiz et al., no “control” concentrations without ECMO were collected so this patient might as well have been an ultra-rapid metabolizer [15]. The first voriconazole concentration obtained under ECMO by Mathieu et al. was supratherapeutic, although a double loading dose was administered [18]. Peterson et al. observed a continuous need for high voriconazole doses under ECMO with only a decreased requirement after decannulation [20], which contradicts the saturation hypothesis [7,11,12,13]. These alternative explanations, along with the insights of our study, question the real impact of ECMO on voriconazole exposure. In our opinion, low voriconazole exposure in these case reports might be attributed to the extensive (non-ECMO related) intrapatient variability and perhaps to publication bias. Our work underscores that case reports and ex vivo studies should be interpreted with caution.

Despite the fact that the overall median voriconazole trough concentration was within target range, a considerable proportion of samples (48%) were subtherapeutic. This is a high proportion but corresponds to the proportions described in two previous published studies in critically ill patients (48% and 53%) [24,25]. In contrast, several other studies reported lower rates of subtherapeutic voriconazole concentrations, around 20%, although in some studies in a population only partially admitted at the ICU [26,27,28,29]. In all the above-mentioned studies, voriconazole trough concentrations were considered subtherapeutic if lower than 1.0 or 1.5 mg/L, while we defined a lower limit of > 2 mg/L. Most guidelines recommend a lower target of 1–2 mg/L, with higher targets (>2 mg/L) for severe infections, treatment of fungi with an elevated MIC value or diseases with a poor prognosis, which may be appropriate in a critically ill population [30,31]. Moreover, a large variability was also observed in the previously published studies, with a reported %CV by Ruiz et al. of 77.7% [24,27].

We acknowledge several limitations to our study. First, this study is limited by its retrospective design. To ensure a correct interpretation of the results, samples that were not collected as trough concentration were excluded from this study. Second, there were only a few voriconazole trough concentrations (*n* = 22) collected during the first three days after ECMO initiation or circuit change. Since the influence of ECMO might be the most prominent during the first ECMO days, due to the absence of saturation at that point [32], most influence on voriconazole is expected in that period. However, the squared day of ECMO was discarded during multivariate analysis, suggesting that this parameter may not significantly affect voriconazole concentrations. Third, in the non-ECMO group, samples from a pre-ECMO and post-ECMO timeframe were combined despite the fact that post-ECMO samples were collected after a longer voriconazole-treatment and possibly escalating doses. Due to the limited number pre-ECMO samples (*n* = 10), no separate analyses were performed; however, in the multivariate analysis covariates such as the day of voriconazole treatment and the voriconazole dose were included. Fourth, as a control group we used voriconazole concentrations from the same patient cohort in the period before or after ECMO treatment, so no external control group was used. Nevertheless, to detect impact of ECMO on voriconazole exposure, it is interesting to control for patient-specific characteristics.

In our study, presenting the largest patient cohort simultaneously treated with voriconazole and ECMO, the extensive intra- and interpatient variability in voriconazole exposure was confirmed [1,16,24,27]. Therapeutic drug monitoring (TDM) is recommended and performed in clinical practice but despite drug monitoring, we still found 48% subtherapeutic samples, which is a lot, certainly in this severely ill population. Isavuconazole and the new formulations of posaconazole might be therapeutic options with a lower variability in exposure [7,33] and were recently shown to be non-inferior to voriconazole for the treatment of IPA [34,35]. For isavuconazole, data still lacks in ECMO patients, with the exception of one case report and one cohort study, in which only a limited influence of ECMO on the isavuconazole concentrations was suggested [36,37]. Concerning posaconazole, an even more lipophilic molecule compared to voriconazole (logP value of 5.5), a prospective study (*n* = 6) recently reported that ECMO did not appear to influence posaconazole exposure, when compared with previously published exposure data in hematology patients [7]. Consequently, these azoles might be justified alternative treatment options in a critically ill ECMO patient population.

## 5. Conclusions

This is the first large, retrospective study investigating the impact of ECMO support on voriconazole systemic exposure. In contrast to previously published ex vivo studies and case reports, this study could not demonstrate an influence of ECMO on voriconazole exposure. This underpins the importance of a cautious interpretation of ex vivo studies and case reports. There was a wide variability in voriconazole trough concentrations and a high proportion of subtherapeutic concentrations in critically ill patients, but ECMO did not significantly contribute to this. TDM of voriconazole remains important, certainly in this severely ill patient population often presenting with subtherapeutic exposure.

## Figures and Tables

**Table 1:** Extracorporeal circuits.

Extracorporeal Membrane Oxygenation (*n* = 69 Patients)	
*Type of ECMO*	
VV-ECMO, *n* (%)	51 (74)
VA-ECMO, *n* (%)	7 (10)
Switch between VV- and VA-ECMO, *n* (%)	11 (16)
*Oxygenator, n (%)*	
Medos HILITE^®^ 7000 LT (Medos Medizintechnik AG)	36 (52)
Medos HILITE^®^ 2400 LT (Medos Medizintechnik AG)	1 (1.5)
Novalung^®^ Heart and Lung Therapy System (Xenios)	1 (1.5)
Cardiohelp^TM^ life support system (Maquet)	1 (1.5)
Quadrox-D oxygenator (Maquet)	2 (3)
Affinity^TM^ oxygenation system (Medtronic)	1 (1.5)
LivaNova ECMO oxygenators (Livanova)	8 (12)
A.L.ONE ECMO oxygenator family (Eurosets)	2 (3)
Unknown	17 (25)
*Bloodpump, n (%)*	
Deltastream DP3 (Medos Medizintechnik AG)	27 (39)
Jostra Rotaflow (Maquet)	6 (9)
Cardiohelp (Maquet)	2 (3)
Biomedicus (Medtronic)	2 (3)
CentriMag (Levitronix)	6 (9)
Revolution sorin (Livanova)	11 (16)
Unknown	15 (22)
*At least one circuit change (or one of its components), n (%)*	22 (31.9)
*Median (IQR) duration ECMO, days*	19 (11–33)
**Continuous Renal Replacement Therapy During Study Period (CRRT) (*n* = 69 Patients)**	
*CRRT, n (%)*	35 (50.7)
CVVH, *n* (%)	31 (88.6)
CVVHDF, *n (%)*	4 (11.4)

*n*: number of patients; IQR: interquartile range; VV: venovenous; VA: venoarterial; CVVH: continuous venovenous hemofiltration; CVVHDF: continuous venovenous hemodiafiltration.

**Table 2:** Voriconazole trough samples.

Sampling Characteristics	Total	ECMO	Non-ECMO	*p*-Value ^c^
Voriconazole Trough Concentrations of Sample Set A ^a^				
Voriconazole Administration and Sampling				
Number of C_min_	282	145	137	NA
Trough concentration (mg/L), *median (IQR)*	2.5 (1.3–4.3)	2.4 (1.2–4.7)	2.5 (1.4–3.9)	0.58
Previous daily dose (mg/kg), *median (IQR)*	8.33 (6.6–11.1)	9.2 (6.7–10.9)	8.1 (6.5–11.1)	0.76
Number of C_min_ per patient, *median (IQR)*	3 (1–5)	2 (1–3)	3 (1–6)	NA
*Severity of illness and type of RRT*				
CRRT_24_, *n (%)*	102 (36)	78 (54)	24 (18)	0.03
IHD_24_, *n (%)*	33 (12)	0 (0)	33 (24)	<0.0001
SOFA score on sampling day, *median (IQR)*	11 (8–15) (*n* = 187)	14 (11–17) (*n* = 90)	8 (6–12) (*n* = 97)	<0.0001
*Variability*				
Inter-subject variability (C_min_/dose) (%CV)	43	47	46	NA
Intra-subject variability (C_min_/dose) (%CV)	73	78	60	NA
Voriconazole trough concentrations of SAMPLE SET B ^b^				
*Voriconazole administration and sampling*				
Number of C_min_	337	190	147	NA
Subtherapeutic C_min_ (<2 mg/L), *n (%)*	163 (48)	106 (56)	57 (39)	0.80
Therapeutic C_min_ (2–5.5 mg/L), *n (%)*	131 (39)	55 (29)	76 (52)	0.37
Supratherapeutic C_min_ (>5.5 mg/L), *n (%)*	43 (13)	29 (15)	14 (10)	0.40
Previous daily dose (mg/kg), *median (IQR)*	8.3 (6.5–10.9)	8.33 (6.6–10.9)	8.0 (6.4–10.7)	0.84
Number of C_min_ per patient, *median (IQR)*	3 (2–6)	2 (1–3)	3 (1–7)	NA
*Severity of illness and type of RRT*				
CRRT_24_, *n (%)*	127 (38)	98 (52)	29 (20)	0.04
IHD_24_, *n (%)*	35 (10)	0 (0)	35 (24)	<0.0001
SOFA score on sampling day, *median (IQR)*	11 (8–15) (*n* = 215)	13 (10–17) (*n* = 110)	8 (6–12) (*n* = 105)	<0.0001
*Variability*				
Inter-subject variability (C_min_/dose) (%CV)	52	59	49	NA
Intra-subject variability (C_min_/dose) (%CV)	78	81	61	NA

C_min_: trough concentration, *n*: number of samples; IQR: interquartile range; CRRT: continuous renal replacement therapy IHD: intermittent hemodialysis; ECMO: extracorporeal membrane oxygenation; ICU: intensive care unit; SOFA: sequential organ failure assessment; CV: Coefficient of variation; ^a^ Continuous: actual trough concentrations, collected 12 h ± 1 h after the previous administered dose; ^b^ Categorical: actual trough concentrations, collected 12 h ± 1 h after the previous administered dose and subtherapeutic concentrations, collected too early (<11 h after previous dose) and supratherapeutic concentrations (>5.5 mg/L), collected too late (>13 h after previous dose); ^c^ *p*-value calculated using univariate GEE analyses with ECMO as binomial outcome variable.

## Data Availability

The data presented in this study are available on request from the corresponding author. The data are not publicly available due to privacy reasons.

## Supplementary Materials

The following are available online at https://www.mdpi.com/article/10.3390/microorganisms9071543/s1, Table S1: Baseline characteristics, Table S2: Distribution of number of C_min_ among different hospitals, Figure S1: Voriconazole trough concentrrations for three different timeframes and S2: Voriconazole dose in function of the day of ECMO.
