# Supplementary material for: A Large Retrospective Assessment of Voriconazole Exposure in Patients Treated with Extracorporeal Membrane Oxygenation
Source: Microorganisms. 2021 Jul 20;9(7):1543. doi: 10.3390/microorganisms9071543 (PMC8303158; doi:10.3390/microorganisms9071543)
Supplement: Supplementary file 1 [file microorganisms-09-01543-s001.zip › microorganisms-1262107-supplementary.pdf]

Supplementary data

Table S1. Baseline characteristics

Patient characteristics (*n*=69 patients)	
Demographics	
Sex, male, *n (%)*	46 (67)
Age, median (IQR), *years*	54 (42–60)
Body weight, median (IQR), *kg*	77 (65–95)
Clinical characteristics	
Length of ICU stay at study site, median (IQR), *days**	40 (26–57)
Deceased during ICU stay, *n (%)*	33 (48)
APACHE II score on ICU admission, median (IQR), (*n*=58)	18 (14–23)
Reason for admission	
Pneumonia, *n (%)*	26 (37.7)
Transplantation, *n (%)*	8 (11.6)
HSCT, *n (%)*	1 (1.5)
SOT, *n (%)*	7 (10.1)
Cystic fibrosis, *n (%)*	5 (7.3)
Sepsis, *n (%)*	5 (7.3)
Dermatomyositis, *n (%)*	5 (7.3)
Interstitial Lung Disease, *n (%)*	1 (1.5)
ARDS, *n (%)*	2 (2.9)
Other, *n (%)*	7 (10.1)
Unknown, *n (%)*	10 (14.5)

n: number of patients; IQR: interquartile range; ICU: intensive care unit; HSCT: Hematopoietic stem-cell transplantation; SOT: solid organ transplantation; ARDS: acute respiratory distress syndrome; APACHE: Acute Physiology and Chronic Health Evaluation

*The ICU stay is defined as the length of stay in the ICU of the participating center. Any stay at ICU, in the period before or after, in another non-participating center, is not mentioned.

Table S2. Distribution of number of C_{min} among different hospitals

C_{min} (*n*=337)	Total	With ECMO	Without ECMO
Hospital 1	2	2	0
Hospital 2	4	4	0
Hospital 3	36	34	2
Hospital 4	37	28	9
Hospital 5	19	14	5
Hospital 6	28	25	3
Hospital 7	209	81	128
Hospital 8	2	2	0

C_{min}: voriconazole trough concentrations; ECMO: extracorporeal membrane oxygenation

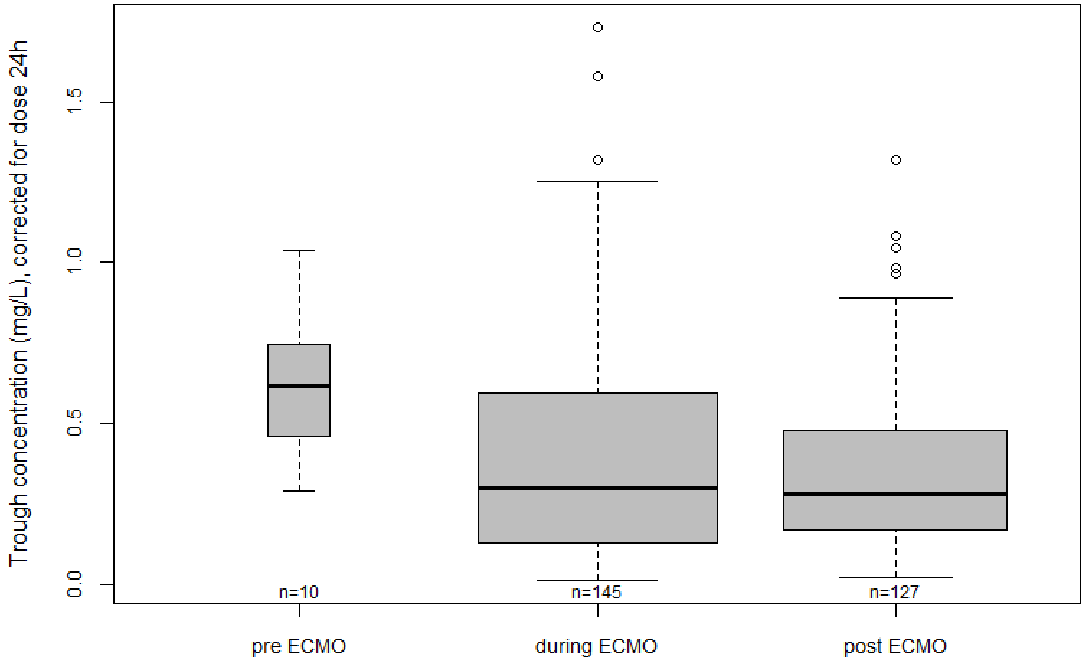

Figure S1. Voriconazole trough concentrations for three different timeframes (pre, during and post ECMO).

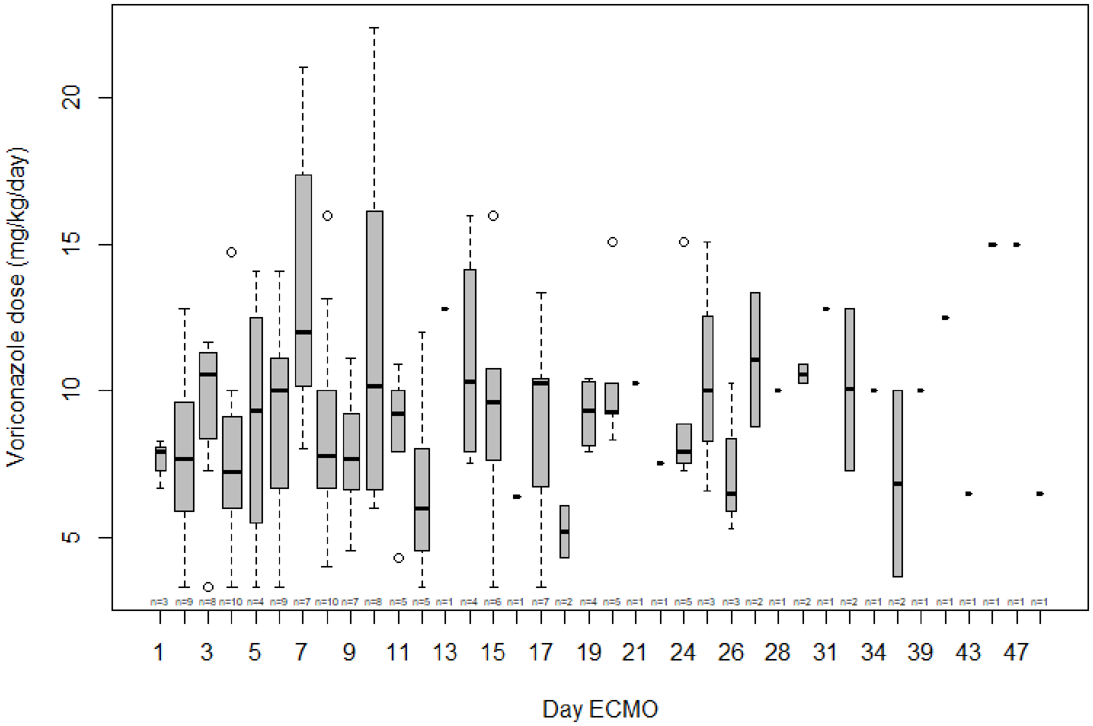

Figure S2. Voriconazole dose in function of day of ECMO.
